# Reviewing the Past, Present, and Future Risks of Pathogens in Ghana and What This Means for Rethinking Infectious Disease Surveillance for Sub-Saharan Africa

**DOI:** 10.1155/2022/4589007

**Published:** 2022-07-14

**Authors:** Peter N-Jonaam Mahama, Amos Tiereyangn Kabo-Bah, Justine I. Blanford, Edmund Ilimoan Yamba, Prince Antwi-Agyei

**Affiliations:** ^1^Department of Civil and Environmental Engineering, School of Engineering, University of Energy and Natural Resources, Sunyani, Ghana; ^2^Department of Earth Observation Science, Faculty of Geo-Information Science and Earth Observation, University of Twente, Enschede, Netherlands; ^3^Department of Physics, Kwame Nkrumah University of Science and Technology, Kumasi, Ghana

## Abstract

The current epidemiological transition makes us wonder how the parallel of infectious diseases (IDs) might be at the end of each passing year. Yet, the surveillance of these IDs continues to focus on high-profile diseases of public health importance without keeping track of the broad spectrum of the IDs we face. Here, we presented the prevalence of the broad spectrum of IDs in Ghana. Data from the annual reports on Gold Coast now Ghana, Global Infectious Diseases and Epidemiology Network (GIDEON), and the District Health Information Management System II (DHIMS2) databases were examined for records of ID prevalence in Ghana. Using the IDs from these databases, the paper assessed the epidemiological transition, pathogen-host interactions, spatiotemporal distribution, transmission routes, and their potential areas of impact in Ghana. The topmost ID recorded in health facilities in Ghana transitioned from yaws in the 1890s to malaria in the 1950s through 2020. We then presented the hosts of a pathogen and the pathogens of a host, the administrative districts where a pathogen was found, and the pathogens found in each district of Ghana. The highest modes of transmission routes were through direct contact for bacteria and airborne or droplet-borne for viral pathogens. From GIDEON, 226 IDs were identified as endemic or potentially endemic in Ghana, with 42% cited in peer-reviewed articles from 2000 to 2020. From the extent of risk of endemic or potentially endemic IDs, Ghana faces a high risk of ID burden that we should be mindful of their changing patterns and should keep track of the state of each of them.

## 1. Introduction

Infectious disease (ID) pathogens form part of the historical streams of life and have successfully exploited the development of human ecology and the evolving natural environment to their advantage. With its changing patterns in a rapidly transformed world, it has become a major problem that poses a persistent threat to global health security and strikes the recognition of the close relationship between human continuous existence, health, prosperity, and stability [1]. According to Berger [2], there are 361 generic IDs in the world, with more than five emerging every year having the potential to spread and become pandemics [3]. Previously remote IDs now compete for global attention in the disease arena and seem destined to play an ever-increasing role in determining disease patterns in ways we could never imagine for our future. Regardless of which pathogen falls under the spotlight, recent patterns of IDs make it plain to us that pathogens do not respect territorial boundaries in our increasingly interconnected world [4]. Some ID pathogens that manifested fully within the past half-century and have transcended into the disease arena to reap global attention include Ebola virus (1970–1979); hepatitis C virus, HIV, and *Escherichia coli* O157:H7 (1980–1989); West Nile virus, Nipah virus, influenza A virus (H5N1), Hendra virus, and hantavirus (1990–1999); Zika virus, SARS-CoV, influenza A virus (H1N1), and *Trypanosoma cruzi* (2000–2009); and *Candida auris*, *Elizabethkingia anopheles* (meningitis), chikungunya virus, MERS-CoV, and SARS-CoV-2 (2010–2020) [5, 6]. The continuous threats posed by these changing disease patterns require local, regional, and global interdisciplinary coordination [7, 8] to develop surveillance systems that can adapt to an increasingly transformed world.

Backed by the recent global outbreak of COVID-19, which overshadowed our strong defence “after emergence” strategy, it reminded us that there is only “One Health” as efforts and policies still fall short of the impetus needed to strengthen interdisciplinary and territorial cooperation for effective disease control and prevention. One Health as defined by the CDC [9] is a collaborative approach from all spheres that integrates the health of humans, animals, plants, and their shared environment. However, global surveillance capabilities and control programs on ID pathogens are critically deficient for the implementation of a global “One Health” system and are mainly challenged by funding [4, 10]. This results in targeting specific geographic areas and diseases after their emergence. According to Stoffel et al. [11], assessing ID outbreaks in isolation is no longer sufficient in our interconnected world. The individual reactive nature of investigations and surveillance programs targeting specific diseases [12], pathogens [13], reservoirs [14], and territorial diversion of attention [15] on different perspectives would most likely render the world more vulnerable to simultaneous outbreaks. Worse of it, about two-thirds of human IDs and almost all known pandemics originate from animals, yet we do not know which zoonosis is the next public health threat. Driven by exponentially increasing anthropogenic changes, these pathogens mainly spill over from wildlife and livestock to humans, resulting in new IDs of pandemic potential [16]. These pandemics and other emerging zoonoses cause us more than two orders of magnitude of annual economic damages apart from the widespread human burden and loss of lives to adapting a One Health surveillance that directs resources to prevent them [3].

In Ghana, just like most Sub-Saharan Africa (SSA) countries, the ability to detect and control emerging and re-emerging infectious diseases (ERIDs) from all fronts is still challenged since it has not yet formalized a national One Health policy [17] and barely account for human health considerations in land-use planning decisions with deficiencies in the veterinary infrastructure, expertise, diagnostic laboratories, and in surveillance capabilities across the country. Thus, the health security of Ghana will continue to be threatened since the preparedness and response capability of a country depend on the availability of such facilities. Successes in antibiotics, immunizations, and the complete eradication of deadly diseases such as smallpox have all been through haven to take decisive stands on a pathogen of concern. Although it is suiting that we can intentionally stand against, control, and possibly eradicate any ID [18] by taking a cue from the smallpox extinction, singling a pathogen as a measure of health crises is just but a drop in the ocean if the entire spectrum of IDs is not considered through a “One Health” system.

The bedrock to the health security of any country through a “One Health” approach should first start locally with a comprehensive and proactive epidemiological approach flanked by an economic willingness to both ID pathogen detection and subsequent interventions. With the rich diversity of animal reservoirs [19] that daily interact with humans such as bats, rodents, birds, pigs, and poultry in Ghana, it will be safer to descend our surveillance on pathogens to the district level by first assessing the distribution and extent of risk of pathogens from this local setting. Thousands if not millions of pathogens from these animal reservoirs are still undiscovered and can infect humans from the rich animal diversity with pandemic potential [3]. However, if we cannot understand and quantify the extent of risk of the existing pathogens, how then do we intend to strategically prepare and control potentially endemic and pandemic pathogens likely to originate from our backyard. These pathogens apart from being devastating enough in their natural state can alter their pathogenic potential by acquiring new genetic combinations that enable them to switch hosts [20]. The synergistic effects of changes in behaviour, socioeconomic inequalities, unsustainable exploitation of the environment, and the ecological characteristics of the hosts all result in the mixing of multiple species to promote pathogens switching hosts. The ability to quantify possible interactions between pathogens and their hosts and pathogens switching hosts can specify the detection and control measures in averting their spread. Rapid detection and response to ERIDs are crucial to averting the rising tide of future diseases since there are more human activities that precipitate pathogens gaining more pathogenic potential and host shift. Although the magnitude of impact might vary when health crises hit, countries of SSA with weak health systems are often ill-prepared and slow to respond to health emergencies compared with strong health systems with possible universal health coverage across the country [21].

If any lessons would be learnt across the centuries, human development although depends on several factors, one of the breakthroughs for SSA would most likely pivot on understanding the epidemiological landscape of IDs and building the capacity to respond, prevent, and transform it into our greatest resource. Its long-term investment losses have always set the region back and equally have the potential to reverse the gains made in our development over the past years as more dangerous diseases continue to emerge and become endemic in the region. Notwithstanding, proactive, real-time surveillance of human, animal, and plant populations for infections will mitigate disease outbreaks and not predictions as research on the resurgence of the Ebola virus in the Democratic Republic of the Congo indicated that no amount of DNA sequencing can tell us when or where the next virus outbreak will appear even with sophisticated machines [22]. It is by this that the past, present, and future risks of pathogens are reviewed to propose rethinking surveillance of IDs to aid the alignment of support, the coordination of efforts, and mutual accountability of research across all spheres. This research, therefore, aimed to assess the epidemiological transition, pathogen-host interactions, spatiotemporal distribution, transmission routes, and their potential areas of impact to understand the risk and epidemiological landscape of the spectrum of IDs in Ghana as a reviewed case study for SSA.

## 2. Study Area, Data Used, and Methods

### 2.1. Study Area

Ghana is a West African country along the Gulf of Guinea with a total area of about 239,000 square kilometres. It is bounded by Togo, Burkina Faso, Côte d'Ivoire, and the Gulf of Guinea. It had an approximate population of 31 million in 2020 [23]. Currently, Ghana has divided administratively into 16 regions with 6 metropolitan districts, 107 municipal districts, and 147 districts, as shown in Figure S1. It has a south-north gradient of population, economic growth, natural resources, land cover, and social conditions [24].

Ghana is located within the arid to humid areas of tropical West African climate and experiences the Intertropical Convergence Zone. Thus, the dry, dusty harmattan trade winds of Northern Sahara and the cool, moist air masses from the Southern Atlantic Ocean come together to form vigorous frontal activities [25]. These activities define the variability of dry and rainy seasons and the amount and duration of rainfalls. This also accounts for the agroecological zones as shown in Figure S2 adapted from [26]: dry northern savannah, the humid middle forest rainfall zone, and the southern savannah and mangroves. Ghana is located close to the Equator and has an average temperature range between 24°C and 30°C [27]. Annual rainfall also ranges from 1,100 mm to 2,000 mm and average humidity of 77% to 85% [27].

Ghana is no exception. The epidemic diseases of any nation reflect the social, economic, and geographical changes. The history of its development based on migration, communications, and rapid urbanization has influenced such a trait. The geographical landscape abundant in rivers and the Volta lake (covering 3% of the country) amasses the presence of prominent vector diseases such as malaria, onchocerciasis, and schistosomiasis [28, 29]. Ghana is still going through an epidemiological transition, so the shared burden of infectious and noncommunicable diseases is now felt [30].

### 2.2. Data Used

Data on ID pathogens in Ghana were obtained from the annual reports on Gold Coast now Ghana, Global Infectious Diseases and Epidemiology Network (GIDEON), and the District Health Information Management System II (DHIMS2) databases (Table 1).

Details for each data set and other health and geographic data obtained are indicated below.

**Figure 1:**
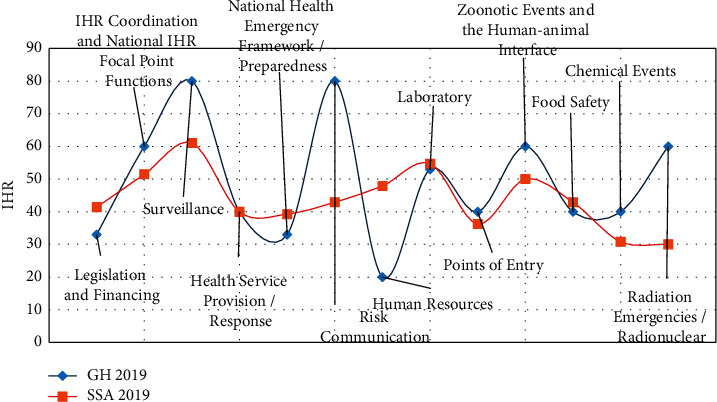
The International Health Regulations (IHR) scores of Ghana and the Sub-Saharan Africa (SSA) region average for 2019.

1. The GIDEON database at the time of the study comprised 361 generic IDs [2]. Of these, 246 IDs are present in Ghana and were used for the study. Out of the 246 IDs, 226 are endemic or potentially endemic in Ghana. Out of the 226 endemic or potentially endemic IDs, 102 of them were studied in 267 peer-reviewed published articles from 101 journals from the year 2000 to 2020 of the GIDEON database. Information extracted from these articles was put into a Microsoft Excel table and included the type of survey for the sampling or study, the infectious disease (ID), month and year of sampling or study, region, district, town, facility, study group, percentage studied or results, month and year the article was published, and journal and title of the article. Three of these articles had their study surveys carried out before the year 2000 but published after the study period. Also, 6 articles had their study surveys started before the year 2000, but the sampling period entered the study period. These two sets of surveys were included in the articles studied. Research articles that included Ghana but covered the whole world, Africa, or the West African subregion were excluded since they were generic and did not specify impacted areas within the country. Case reports, sporadic case reports, outbreak reports, cross-border reports, and Program for Monitoring Emerging Diseases (ProMed) reports were also excluded since they were not peer-reviewed articles. From the 267 peer-reviewed published articles, 1,388 district survey sites were extracted. Out of the 1,388 district survey sites, 1,099 were obtained directly after reading through the articles, 279 sites were obtained by georeferencing maps produced in the articles, and 10 sites were obtained by contacting their article authors through electronic mail.
2. Data on pathogen-host and related interactions for Ghana were extracted from the SpeciesInteraction_EID2 database [31]. Based on evidence publications and nucleotide sequences, the SpeciesInteraction_EID2 database provides information on all possible pathogens of a host and all the hosts to a pathogen. The IDs' names and their synonyms as presented by GIDEON were used as a reference to identify all possible hosts of a pathogen from the SpeciesInteraction_EID2 database that can be attributed to Ghana. Thus, taking the name of an ID from GIDEON, the host(s) was then searched from the SpeciesInteraction_EID2 database. Out of 246 IDs, 245 had their host in the SpeciesInteraction_EID2 database and extracted.
3. International Health Regulations (IHR [2005]) is a monitoring tool by the WHO to assess the implementation of the core public health capacities in averting the international spread of diseases. About 196 participating countries, therefore, report their yearly performance on the 13 core capacities indicated in Figure 1.
4. Infectious diseases reported through the District Health Information Management System II (DHIMS2) were obtained from the Policy, Planning, Monitoring, and Evaluation Division (PPMED) of the Ghana Health Service (GHS). This consists of case count data (laboratory-confirmed and presumed) for IDs from 9,373 healthcare facilities across the country that were reported through DHIMS2 at the period the data were obtained. The DHIMS2 started collecting and collating its routine health service data for health facilities in 2012 to date. The data used for the study analysis were 3 IDs (typhoid fever, malaria, and influenza-like illness) from January to December 2020.
5. The Facts and Figures of the GHS provide a yearly overview of the health sector's performance. This provides the topmost 20 causes of outpatient morbidity reported at health facilities, which were available for the period 2002 to 2017. Three of the topmost IDs identified from the Facts and Figures were typhoid fever, malaria, and influenza-like illness based on their type of pathogen; these IDs are caused by bacteria, parasites, and viruses, respectively, as the selected pathogens for further assessment.
6. Annual reports on the Gold Coast now Ghana are colonial reports that highlight the performance of each sector of the country. Data on IDs from the health section of these documents were obtained from 1895 to 1954. The target information was the IDs that dominated within that period and their transition.
7. Additional data sets that capture other geographic information such as demography and administrative boundaries of Ghana are described below.
8. The Ghana Statistical Service (GSS) keeps data on the demography of the country. The 260 district names were obtained from the GSS.
9. The administrative boundaries of the districts were obtained from City Population [32]. Shapefiles for 44 districts were obtained by georeferencing the administrative boundaries from City Population and subsequently digitized from the existing 216 available shapefiles to obtain the complete 260 districts' shapefiles used to develop maps of the study.

### 2.3. Analysis of Data

The analysis was based on the prevalence of IDs in Ghana, which was presented in two phases: first, the highest reported IDs from 1895 to 2020; and second, the broad spectrum of IDs from 2000 to 2020. The second phase was to study the current extent of the risk of IDs in Ghana. To understand the epidemiology of these IDs, we need to identify their pathogens, hosts, and methods of transmission.

#### 2.3.1. Epidemiological Transition of Ghana

The annual reports of the Gold Coast now Ghana were reviewed for information on ID prevalence by going through the health section of the reports from 1895 to 1954. Tuberculosis, malaria, trypanosomiasis, yaws, diseases of the enteric group, smallpox, cerebrospinal meningitis, leprosy, venereal diseases, yellow fever, helminthic diseases, nephritis, and respiratory infections were some of the principal diseases prevalent in the Gold Coast. However, the ID with the highest cases reported at health facilities for each year of the reports was identified. This was used to assess the epidemiological transition of the most endemic ID and other epidemics that occurred over that period. With the lack of the annual reports after 1954, beyond this year were reviews of documents of the GHS, the Ministry of Health, and relevant peer-reviewed literature. This was to identify the highest ID reported after 1954. The years in which some global pandemics reached Ghana were also identified.

#### 2.3.2. Current Extent of Risk

Some global databases presented in the peer-reviewed literature from 1940 to 2020 contained vital information on the position of Ghana to the risk of ID prevalence. This can be parameterised into Ghana being known for emerging infectious diseases (EIDs), parasite richness, undiscovered parasite diversity and predicted risk, and rodent reservoirs for future zoonotic diseases. On the scale of low to high, it was assessed to see at which position Ghana was placed for the extent of risk for these parameters. Further, using the broad spectrum of IDs from 2000 to 2020, a number of endemic or potentially endemic IDs extracted from the GIDEON database were reviewed for their extent of risk. Using these data and supplemented by other data, the risk of pathogen-host and related interactions and pathogen distribution and transmission were assessed as described below.

*(1) Risk of Pathogen-Host and Related Interactions*. Pathogens are the cause of any ID. The type of pathogen of each ID in Ghana from the GIDEON database was identified and extracted into bacteria, virus, parasite, fungus, protoctista, and unknown. Using the name and/or synonyms of an ID, it was then cross-checked from the SpeciesInteraction_EID2 database [31] for the reservoir(s) that hosts each pathogen. Not based on any literature criteria, the 21 classified reservoirs of the SpeciesInteraction_EID2 database were recategorised into 17 reservoirs. The IDs of each type of pathogen were then summed for each of the classified reservoirs. This formed the number of IDs for each reservoir and pathogen. The percentage of the pathogens per each category of the reservoir was then calculated. Using comparative and descriptive approaches [33], the pathogen-host interaction and the species at high risk of transmission of infections were described.

*(2) Risk of Pathogens Distribution*. The spread of a pathogen is facilitated by an infected individual or vector moving through the susceptible population and might move to larger geographic regions. The IDs of 1,388 district surveys extracted from GIDEON were used to create a map to show the spatial distribution of pathogens from the year 2000 to 2020. The distribution of the map depended on the number of districts surveyed. Also, the intensity of the map depended on the number of surveys per district.

Further, the geographic distribution of pathogens from the DHIMS2 data was presented as spatial maps based on the cases recorded from the 9,373 healthcare facilities. From January to December of 2020, cases of typhoid fever, malaria, and influenza-like illness were summed for each of the facilities. The facilities within each district were also summed to develop the spatial map of the respective pathogens of the IDs for each district.

*(3) Risk of Pathogens Transmission*. The route of transmission of pathogens shows how IDs are spread to new hosts and geographical locations. The interplay between a pathogen, the host, and its route of transmission thus serves as the causation on which the spread of any ID is centred. Humans, animals (companion, production, and wildlife), plants, and inanimate matter in the environment serve as reservoirs and equally as the source through which the transmission routes of IDs exist. Since some pathogens can exist in more than one type of reservoir, such pathogens have different transmission routes.

Although several classification schemes are used, the most common forms of categorising transmission routes have been direct and indirect transmission. Direct transmission involves the infective form of a pathogen transferred directly from a reservoir to an infected host, while the indirect transmission takes place through a live or inanimate intermediary [34]. The risk of pathogens transmission of the 246 IDs in Ghana from the GIDEON database was categorised into direct contact, airborne or droplet-borne, fomite, none, and unknown as specified by GIDEON and are as shown in Table 2.

The route of transmission as specified by GIDEON was confirmed by crosschecking information from the Centre for Disease Prevention and Control [35] and the European Centre for Disease Prevention and Control [36]. Each pathogen was categorised on how it is transmitted. Multiple routes of ID transmission were identified. Pathogens of multiple routes of transmission were put into each of their respective categories. The number of pathogens for each transmission route was then summed.

## 3. Results

### 3.1. Epidemiological Transition of Ghana

Data from Ghana (the ex-Gold Coast) annual reports indicated the presence of diseases, including yaws, malaria, gonorrhoea, pneumonia, tuberculosis, influenza, syphilis, and other tropical diseases. Because these diseases were not presented in any chronological manner in the annual reports and subsequent documents investigated, no graphical layout could be used to demonstrate the transition of IDs over the years. However, yaws and malaria were seemingly holoendemic in the first half of the 20^th^ century, with the former constituting as high as 65% and the latter as high as 27% of all IDs reported. Furthermore, there were no records of publication of medical data and the annual reports after 1954 [37], it can be ascertained that malaria flipped over the coin as the highest reported case in the late 1950s and thereafter increased steadily. In terms of mortality, respiratory diseases, tuberculosis, and malaria have been at the epic from the records of the annual reports.

Unlike the top IDs reported annually over the years, some of the deadliest epidemics in history that caught global attention had also been reported in Ghana, as shown in Table 3.

The influenza pandemic of 1889–1893 reached the shores of the Gold Coast (called now Ghana) in 1891 with subsequent annual cases [38]. The bubonic plague, which was believed to be introduced into the country by infected rats in cargos, got to the ports of Accra and Sekondi in 1908 and 1924, respectively [39]. The influenza epidemic of 1918–1919 [40], which reached the Gold Coast through shipping and overland travellers, was devastating. The colonial annual report for 1918 placed the total mortality as 30,000, assuming three-quarters of the population were affected [43]. Other accounts place the total mortality as over 100,000 deaths in less than six months [44]. This implies that 2% to over 5% of the population was lost to the influenza epidemic. HIV/AIDS was first reported in 1982 and has since then persisted until perhaps a cure is found [41]. SARS-CoV-2 was reported in 2020 and has since caused great havoc and fear for the future as cases are still recorded after two years of reaching Ghana [42].

### 3.2. Current Extent of Risk

From a global perspective, Ghana is placed under certain categories from peer-reviewed databases. Based on a global database [45] since 1940, Ghana falls within the moderate-to-high-risk zone for all known EIDs [46] that have changed patterns over time. Based on the Global Mammal Parasite Database, Ghana falls within the moderate to high risk in parasite richness, undiscovered parasite diversity, and predicted risk [47]. Similar research indicates that Ghana has a moderate to high risk of rodent reservoirs for future zoonotic diseases [48]. A great range of the country's geographical cover is predicted to host *Rousettus aegyptiacus* bat species that are positive for the Nipah virus from a global study [49]. The country is also depicted as having a geographic range of 29 primate species with a high-risk score for testing positive for the Zika virus [50].

Moreover, based on the GIDEON database, the average number of endemic or potentially endemic IDs was 226 for Ghana and 222 for SSA; Ghana's neighbouring countries Burkina Faso, Cote d'Ivoire, and Togo had 226, 233, and 221, respectively. From the 2019 review of the Global Health Security Index, Ghana scored 35.5% compared with the average of 30.85% of the SSA region. The result of the IHR as of 2019 for Ghana compared with SSA is shown in Figure 1. Further, on confirming the first case of COVID-19 on March 12, 2020, Ghana had 88,228 cases and 698 deaths in one year, and 161,761 cases and 1,445 deaths two years after [51]. This made Ghana fall within 80 to 115 on the global chart and within 5 to 10 of the SSA region in terms of the number of cases. The cases and death over the first two years seem similar each year with four peaks of the virus within two years in the country.

#### 3.2.1. Risk of Pathogen-Host and Related Interactions

Of the global generic IDs recognised by the GIDEON database, 63% are said to be endemic or potentially endemic in Ghana. The interaction between the type of ID pathogens and their reservoirs is summarized as percentages in Table 4.

From the results, out of 246 pathogens identified in Ghana, humans serve as a reservoir for 92.7% of these pathogens. Apart from the human burden that is always quantified, approximately 71.5%, 47.2%, and 30.8% of these human pathogens can affect domestic animals, mammals (excluding humans), and primates (excluding humans), respectively, by reverse zoonoses. 17.0% of these human pathogens can also possibly affect plant health. About 0.05% of these IDs in Ghana can be classified as neglected tropical diseases.

#### 3.2.2. Risk of Pathogens Distribution

**Figure 2:**
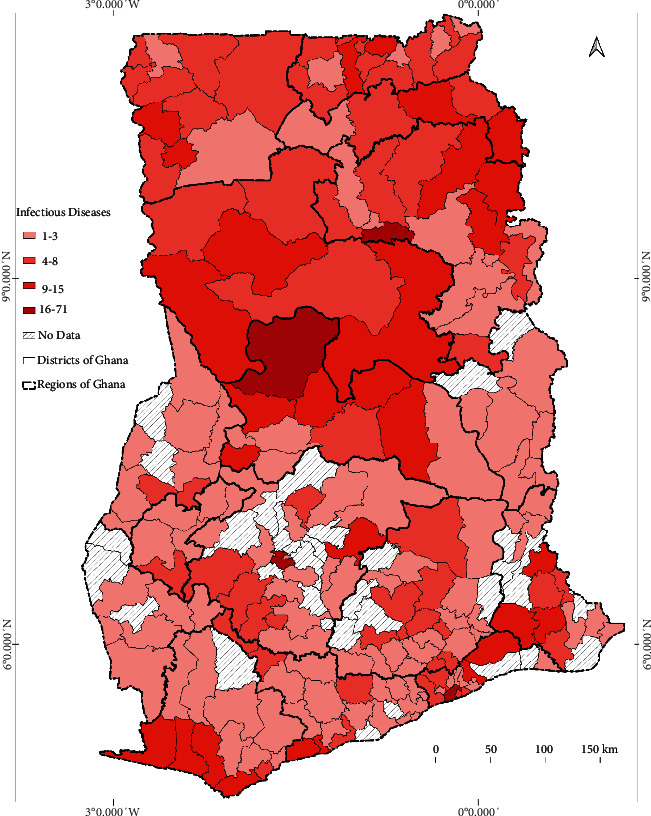
Distribution and intensity of endemic or potentially endemic infectious diseases studied in Ghana from 2000 to 2020. The legend indicates the number of infectious diseases studied in each district across the country. For instance, 1 was the least number of infectious diseases studied in a given district and 71 was the highest number studied. The intensity classifications were generated based on quantile to assign the same number of data values to each class from the ArcGIS software used to develop the map.

From the GIDEON database, 42% of IDs in Ghana were studied with peer-reviewed publications from 2000 to 2020 as prevalence or seroprevalence surveys. The intensity and distribution of the studied ID pathogens across the country are shown in Figure 2.

**Figure 3:**
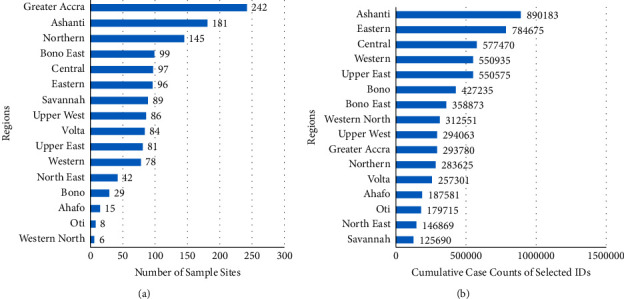
(a) The regional breakdown of the peer-reviewed articles on surveillance of the spectrum of IDs in Ghana. (b) The regional breakdown of cumulative case counts of selected IDs across the country. These IDs are the highest reported cases of their pathogens at health facilities and include typhoid fever, malaria, and influenza-like illness.

The Greater Accra municipality recorded the highest number of studied IDs. A regional and district breakdown of the results is shown in Figure 3(a) and Table 5. This indicates that the percentage of individually studied pathogen groups ranged from 0 to 61%. The highest studied pathogen group was parasites, and the lowest was protoctista and unknown pathogens. Elaborating the table for instance, out of 106 bacterial pathogens known in Ghana, 29 were studied over the period, and 12 were sampled from 54 districts of 125 different time frames at the district level. Unknown and protoctista pathogens were relatively disregarded over the twenty years. The number of pathogens studied was more at the metropolitan than the municipal and district levels. However, the number of areas studied and the number of times the studies were carried out were more at the municipal and district levels compared with the metropolitan level.

From the facts and figures of the GHS, the topmost twenty causes of outpatient morbidity reported at healthcare facilities from 2002 to 2017 are associated with ID pathogens constituting about 70%. These include malaria, upper respiratory tract infections, diarrhoeal diseases, skin diseases, acute eye infections, intestinal worms, acute ear infections, typhoid fever, pneumonia, vaginal discharges, acute urinary tract infections, septicaemia, otitis media, and chickenpox. Out of these, 29% are caused by bacteria, 12% by viruses, 15% by parasites, and 15% by fungi.

**Figure 4:**
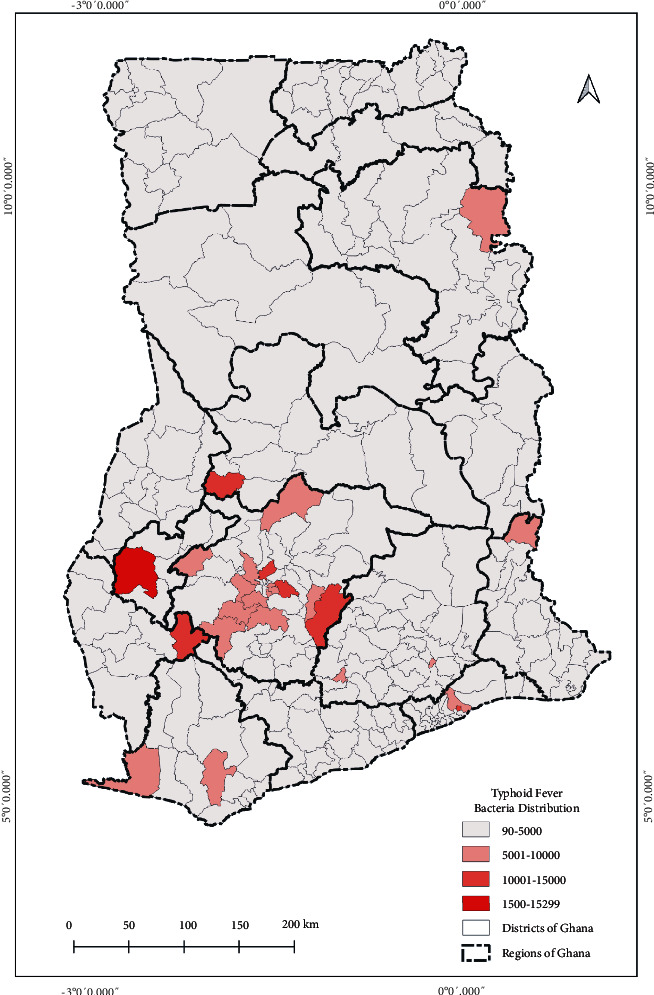
Distribution and intensity of typhoid fever bacteria pathogen in Ghana for the year 2020. The legend indicates the sum of typhoid fever cases recorded from health facilities in each district across the country. For instance, 90 was the least number of cases recorded from a district and 15,299 was the highest number of cases recorded from a district within the period of January to December 2020. The intensity classifications were generated based on quantile to assign the same number of data values to each class from the ArcGIS software used to develop the map.

**Figure 5:**
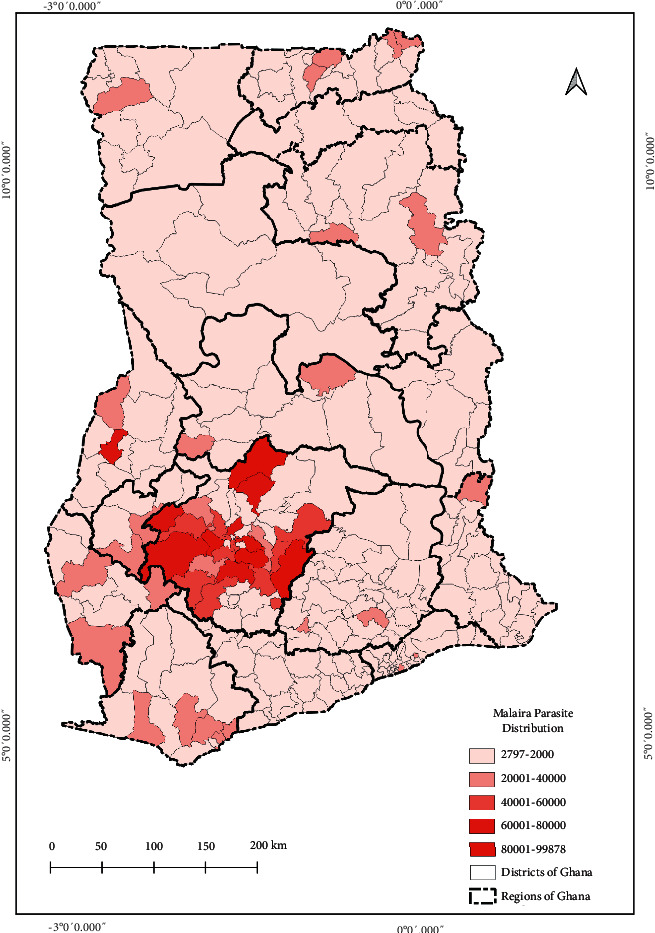
Distribution and intensity of malaria parasite pathogens in Ghana for the year 2020. The legend indicates the sum of malaria cases recorded from health facilities in each district across the country. For instance, 2,797 was the least number of cases recorded from a district and 99,878 was the highest number of cases recorded from a district within the period of January to December 2020. The intensity classifications were generated based on quantile to assign the same number of data values to each class from the ArcGIS software used to develop the map.

**Figure 6:**
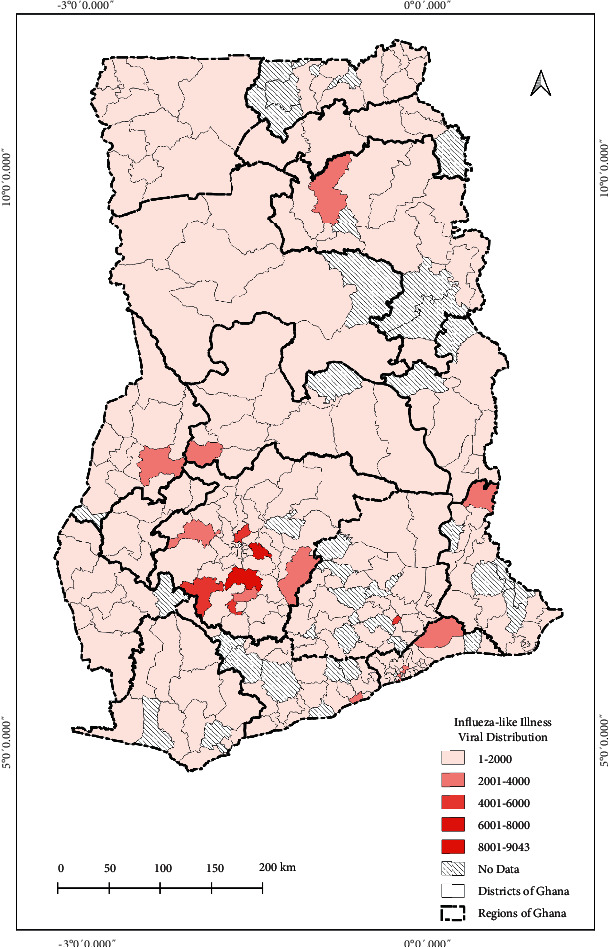
Distribution and intensity of influenza-like illness viral pathogens in Ghana for the year 2020. The legend indicates the sum of influenza-like illness cases recorded from health facilities in each district across the country. For instance, 1 was the least number of cases recorded from a district and 9,043 was the highest number of cases recorded from a district within the period of January to December 2020. The intensity classifications were generated based on quantile to assign the same number of data values to each class from the ArcGIS software used to develop the map.

The current state of pathogens' spatial distribution of selected bacteria (typhoid fever), parasite (malaria), and virus (influenza-like-illness) infection cases at healthcare facilities across the country reported through DHIMS2 for the year 2020 is shown in Figures 4–6, respectively.

The distribution indicates the total number of cases reported within each district. From the maps, although the pathogen's distribution can be described as relatively homogeneous, the Ashanti Region recorded the highest distribution of cases as shown in Figure 3(b).

#### 3.2.3. Risk of Pathogens Transmission

**Figure 7:**
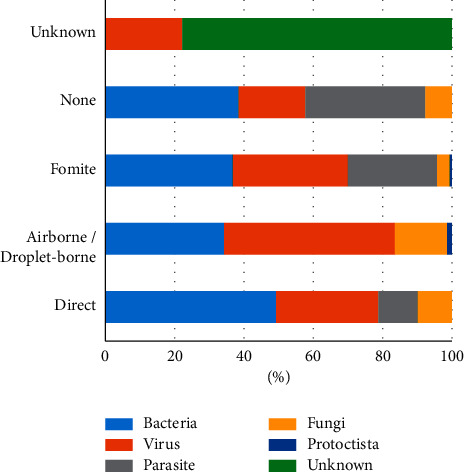
The transmission routes of the infectious disease pathogens in Ghana. This indicates the transmission routes and the pathogens associated with them in percentages.

The transmission routes of ID pathogens in Ghana are shown in Figure 7. The analysis of this figure indicates that transmissions through direct contact are more associated with bacteria (49%) followed by viruses (30%). Transmissions through airborne or droplet-borne are more associated with viruses (49%) followed by bacteria (34%). Transmissions through fomite are more associated with bacteria (36%) followed by viruses (33%). Transmissions through none of the defined routes are more associated with bacteria (39%) followed by parasites (35%). Transmissions through unknown routes are associated with unknown pathogens (78%) and viruses (22%).

## 4. Discussion

### 4.1. Epidemiological Transition of Ghana

Unlike the Western and the Mediterranean world, where the advanced art of writing left impressive documentary records that served as evidence to translate the hierarchies of diseases over centuries, not much can be said about Ghana. Even with the absence of documented evidence, Ghana in the African continent could not have missed the fundamental footprints of ID changes that shaped the history of the world. There could have been several diseases rooted in the rich zoonotic biodiversity of the tropics and the presence of disease vectors and those introduced by human interaction, movement, and external contacts of subject populations of Africans even before the Atlantic Trade with the Western world. Smallpox, measles, and plague were relatively familiar to the African continent with some believing that they even originated from here [52].

In Ghana, some archaeological evidence indicated that thriving settlements believed to have been formed in the second half of the first millennium Common Era and abandoned in the middle of the second millennium, which being related to the brutal waves of IDs such as plague [53]. Recall from oral traditions also accounts for abandonment and resettlement of communities due to the outbreak of diseases such as smallpox [54] and other IDs accompanying human movement and resettlement [55] in the 19^th^ century.

The epidemiological changes in disease patterns as captured by Mensah and Aikins [56] transitioned from the “Age of Pestilence and Famine” due to the shift in agriculture to the “Age of Degenerative and Man-Made Diseases” characterised by a decline in IDs based on Omran's model [57]. Currently, the evolutionary story we have resided over a few decades down the line is the “Age of Emergent and Re-emergent Infections” with evidence of infectious and nutritional diseases of the agricultural shift age, spikes of IDs previously thought to be toppled over by degenerative diseases coupled with new diseases [58]. This can be attributed to the current global human advancement and microbial adaptation. In the face of climate change, globalization, and the adaptive trend of IDs, the age of emerging and re-emerging infectious diseases (ERIDs) is far to be closed if eradication measures are not adaptable to these changes [59]. The transition of diseases in Ghana, as in most developing countries, is still bedridden with unhygienic environmental conditions; weak public health systems and medical care are yet to see the full scale of the epidemiological transition witnessed by the developed countries. This is because IDs still rule the burden of morbidity and mortality in Ghana. With the current trend of diseases, the transition into Omran's second model stage, the “Age of Receding Pandemics” might just be part of the history told of the developed world. Ghana experiences a double burden of diseases; infectious and noncommunicable diseases [30, 60]. Although historical records indicate the presence of chronic diseases in the 19^th^ century with the first hospital recording a case of stroke from the Korle Bu hospital in the 1920s [61], the medical front has been dominated by IDs to the present age. Ghana through its epidemiological transition from 1895 to 2020 was determined to have been holoendemic to malaria and yaws with the latter being flipped over by the former as the highest recorded cases. Ghana now accounts for 3% of the global malaria burden and is among the top 15 countries [62]. In 1951, the trypanosomiasis/yaws campaign services were redesignated into medical field units with a wider scope of mass treatment campaigns and services that covered more IDs [63]. However, due to lack of information during that period, it could not be ascertained what caused the flip of the highest recorded ID from yaws to malaria apart from the mass treatment campaigns. Although the number of yaws cases has reduced significantly from the late 1950s to date, Ghana still serves as one of the countries with the most cases in the world. This West African country is one of three countries (including Papua New Guinea and Solomon Island) that accounts for 84% of global yaws cases [64].

### 4.2. Current Extent of Risk

From the early attempts of the public health reforms in 1878 such as the first public health law [65] and ban on traditional healing [66], the country Gold Coast now Ghana has failed to shift its policy emphasis from curative to preventive even after achieving some testimonies from yaws campaigns. This could account for the cause of our dominant preventable ID burden in the present century. The mobile population of that time enabled the homogeneous epidemiological units of pathogens and vectors across the country. Slave deportation, World War I and II, and other geopolitical factors [56] also saw the immigration of a lot of people in the country who could have brought with them their recognised diseases apart from the unsanitary conditions such immigrants create to escalate disease proliferation. One thing for sure is as we continue to disturb the natural habitat and push animal vectors closer to the human population, we will be threatened with more ERIDs.

The verified global databases indicated the moderate-to-high-risk zone within which Ghana falls for all known ERIDs. This could be a result of the geographical zone and demographic diversity of the country (Figures S1 and S2). The number of endemic or potentially endemic IDs, the GHSI scores, and the IHR for Ghana compared with SSA can be approximated to fall within the same respective ranges. The pattern, distribution, burden, and description of the ID pathogens in Ghana can be argued to reflect that of most SSA countries. Therefore, Ghana can be used as a benchmark for surveillance and to assess the performance of the SSA region in terms of IDs for the present age.

#### 4.2.1. Risk of Pathogen-Host and Related Interactions

The ecological communities of humans, animals, plants, and the environment form an interaction. In the same vein, pathogens can also form complex ecological communities of interacting organisms within their host [67]. From the results, we presented all the hosts of a pathogen and the pathogens of a host in Ghana. It was observed that by reverse zoonoses, approximately 70% of human pathogens can infect other animal hosts, a burden always not quantified. Recent research indicates the striking frequency of the emergence of ID pathogens as unprecedented with major pandemics originating from zoonoses. Generally, 60% of all human pathogens and about 75% of EID pathogens are zoonotic, of which 72% are from wildlife with cross-species transmission capabilities [6, 68].

In terms of animal species, our research findings indicated that human pathogens possibly affect domestic animals more than other animals. This could be due to the increase in human exposure and contact because of human closeness to these animals. Therefore, it is not surprising that many human pathogens are shared with close animals. However, the risk of accidental transmission of pathogens that have not yet crossed the species barrier represents the looming threat of intense surveillance because of the growing exposure of humans and these animal contacts [69]. The number and diversity of reservoir species of a pathogen determine its capability to shift host species, its period of retention in the disease arena, and possible spread to other geographical locations. Research indicates that the shifting of reservoir species of pathogens can also be linked with environmental and climatic conditions that provide the opportunity for pathogens to be exposed to new host species, its spill-over, and subsequent spread [70]. The level of transmission risk between species also depends on the interaction between these species. For the emergence and transmission of zoonotic pathogens, human actions dictate the level of risk [71]. Undoubtedly, exposure to animal species close to the human environment increases the risk of cross-species transmission and pathogens spilling over into new host populations. From the results, it was also established that it is not all neglected disease pathogens that are neglected. Although, some of them have attracted more attention and in-depth studies than some traditional disease pathogens.

The enhanced use of information to understand multiple agents, simultaneous infections, and species interaction is significant to anchor the importance of health security and evolving issues of ID pathogens. It enables us to understand the biological mechanisms to host vulnerability, the immune response to these infections, public health burdens, and prevention and control of these infections. Thus, building the synergy between disciplines will reduce the collective burden of diseases, improve health and livelihoods, and prevent cross-species disease transmission.

#### 4.2.2. Risk of Pathogens Distribution

To our best knowledge, this study is the first to explore the spatiotemporal distribution of all ID pathogens in Ghana. The coordination of activities, surveillance, and investment can improve conditions for national health security. However, the responsibility lies at the district level before it progresses to the national level. From the results, we presented all the districts where pathogens were studied and all the pathogens studied in each district. In Ghana, among the many ID pathogens, some have progressively declined, while some such as Guinea worm, human African trypanosomiasis, and trachoma recently have been eliminated [29]. A potent effect of such elimination and reduction in pathogenic potential is translating to improved economic development [29].

Notwithstanding, no disease pathogen should be left to chance since we do not know where and when the perfecting storms of these pathogens may strike. In Ghana, malaria for instance has become the topmost reported case at healthcare facilities, endemic, drug resistant, adaptive to climatic changes, and a stark reminder of the systemic failings of response mechanisms and the inability on the part of decision-makers to predict and insight early warnings of outbreaks [72, 73]. Also, some re-emerged diseases outbreak once thought to have been dormant or eliminated end up re-emerging and becoming more disastrous than ever. In Ghana, the likes of Poliovirus last reported in November 2008 resurfaced in August 2019 after a decade [74].

The size of the host population influences a pathogen's life cycle as it determines the limits of spread. If a population (particularly the population of susceptible individuals) is not sufficiently large, the disease will die out after infecting all possible hosts. If the population is large enough, the disease can persist until births or migration provides access to new hosts. An increase in the human population and the interactive nature of the Ghanaian societies make it possible to witness the share size of the persistent pathogenic potential of human infections. From the results (Figure S1), 44.5% of the demographic administrative divides are of the municipality and metropolitan status. An additional 19.6% of the districts are projected to have municipality population status [32]. This makes a total of over 64% of the Ghanaian district population obtain a municipal status. This population has not only impacted human exposure and transmission capabilities but also animals and their shared environment. For the seemingly homogeneous disease pattern, selling of diseased animals for consumption by farmers to recover losses not only in the rural areas but also in the urban areas can also acclaim to the effect. Our wildlife has also served as a legal commodity for income and protein, especially for rural folks or transported to urban markets for higher prices with some forests being depleted of such resources [75]. The clearing of forest reserves for wood, agriculture, and mining creates a geographic overlap of humans, wildlife, and domestic animals. Our forest reserve has declined with the deforestation rate paged at 0.6% per year [76].

The seemingly homogeneous south-north gradient disease pattern as seen in the resulted maps can thus be said to be attributed mainly to the similar population density and social preference characterised by the country. However, rapidly growing population areas are causing an increase in contact rates among individuals. In the Greater Accra Region, Accra, the capital city with the highest population, had most of the pathogens studied (Figure 2). However, the distribution of the healthcare facilities' ID pathogens for the year 2020 indicated that the intensity of the three major IDs was concentrated in the Ashanti Region, as shown in Figures 4–6. We presented hotspot areas where species are at risk of host shift from humans, domestic animals, and wildlife from the maps. We established that because of the tropical nature of the humid forest zone (Figure S2), those areas form a hotspot to host shift [77], evolution, and transmission of novel zoonotic pathogens from wildlife. However, the savannah zones will be more vulnerable to host shift, evolution, and transmission of novel zoonotic pathogens from domestic animals due to their suitable free-ranging demographic nature for rearing animals.

#### 4.2.3. Risk of Pathogens Transmission

The interaction and behaviour of hosts and pathogen vectors determine the dynamics to which IDs spread. The movement of hosts and pathogen vectors can be described as the leading cause of ID transmission. The behaviour in response to the perceived risk of infection also degenerates into IDs spread. The interaction and behaviour determine the effectiveness of a given route to transmit pathogens. Thus, the pathogenic potential of any pathogen depends on the vehicle of transmission. In most realistic cases, the pathogen with successful human-to-human transmission coupled with multiple routes is a dangerous pathogen to avoid.

The recent COVID-19 pathogen-host interaction has made us appreciate on a global scale how an infection can move from one animal species to humans, and then from human to other animal species, through a variety of compromised transmission barriers [78]. For a country with weak health systems, it is a risk to have 49% of viral and bacterial pathogens having the ability to be transmitted through direct and airborne or droplet-borne knowing how porous our societies are. From the results, it is even riskier to have unknown pathogens that we do not know their transmission routes constituting 78% of such pathogens. Therefore, it would be logical to rethink the public health surveillance system to cut across expertise and simultaneously assess all IDs and species since our transmission barriers are being overcome and exposures increase.

### 4.3. Sentinel Sites for Integrated Disease Surveillance Implications for Policy and Research

About 226 of the global ID spectra are said to be endemic or potentially endemic in Ghana as of 2020. While the list of IDs is growing steadily, the risk of infection is exploding as well. Improvements in the taxonomic designations of infectious organisms, the availability of highly advanced reference laboratories capable of accurately identifying infectious organisms, increases in the number of immune-compromised patients susceptible to infections by organisms that are not otherwise pathogenic, and the ease with which infections can be transported from place to place throughout the modern world have all contributed to the increase in newly encountered rare IDs [79]. As the world grows to greater heights, the standards for planning and evaluation of interventions for health security are more demanding. The collaborative approach to disease surveillance in anticipation of pathogen spill-over events for different reservoirs should be well anchored through a network of sentinel sites to strengthen existing capacities. The use of such a system should attempt to sample humans and other reservoirs, especially of the rural but more vulnerable populations that do not have access to or attend healthcare facilities. One eminent issue in our health system is the fact that we turn to be treatment bias than be proactive in finding the determinant of a patient's health. The diagnoses of diseases are sometimes based on clinical intuitions than laboratory confirmation. For instance, fever is often underdiagnosed and intuitively treated as malaria. The healthcare facilities alone are not able to give a complete picture of the ID pathogen burden. The norm of disease surveillance that cuts across Ghana and the SSA region has also been disease-specific or targeted groups. Thus, the complete neglect or denial of the existence of some diseases would turn to create the proliferation or perfecting of their storms.

By looking at the epidemiological priorities of each district as obtained from the results, we should be able to team up with their public health needs for effective decision-making. For instance, areas within the meningitis belt (that is, the northern zone of Ghana) are yearly struck with the disease, yet it does not fall within the actionable national priorities. The emergence and re-emergence of ID demand we rethink our surveillance system to fit into recent research trends and capacities without neglecting certain demographic areas or pathogens. Such a system as part of future studies would be able to keep track of the pathogenicity and virulence of pathogens, map areas where new diseases emerge, keep track of the human demographic trend that contributes to pathogens abundance, and quantify the effects of pathogens on health security. However, the lack of coordination between human, animal, and plant surveillance systems [80] plays a great role in hindering the effectiveness of such a “One Health” concept in averting the spread of ID pathogens. The coordination of surveillance systems across expert fields would help identify EID threats at the source and find solutions from the perspective of bridged disciplines. Beyond the synergistic effects through coordination, rethinking our surveillance strategy through “One Health” [69] will enable us to understand and create awareness of the potential for ERIDs while creating the necessary atmosphere for the control of its perfecting storms.

## 5. Conclusion

The study “Reviewing the past, present, and future risks of pathogens in Ghana and what this means for rethinking infectious disease surveillance for Sub-Saharan Africa” assessed epidemiological transition, pathogen-host interactions, spatiotemporal distribution, transmission routes, and their potential areas of impact in Ghana based on data from various sources including Global Infectious Disease and Epidemiology Online Network, SpeciesInteraction_EID2 database, International Health Regulations, and District Health Information Management System II. The study determined that 226 endemic or potentially endemic infectious diseases have been reported in Ghana and 222 on average for SSA. Humans served as the biggest reservoir of disease pathogens (92.7%), and transmission through direct contact, fomite, and none was more associated with bacteria, while airborne or droplet borne and unknown was more associated with viral pathogens. Unknown pathogens with about 78% of their transmission routes also unknown put the population on a time bomb. It was ascertained that indeed Ghana faces a high risk of ID burden and that we should be mindful of their changing patterns and keep track of the state of each of them. Else, climate and geographical changes may just be warming up the extent of epidemics we witness as more threats confirm that most IDs originate and are sustained from host shifting as a result of these changes. Relying on the concept of “One Health” across the spectrum of the pathogen dynamics in Ghana, this research should push the debate on restructuring surveillance strategies to be able to tackle the limitations of this paper. For instance, the cause of the highest recorded ID in Ghana flipped from yaws to malaria in the late 1950s, quantifying the burden of human pathogens on other animal species and the extent to which a given transmission route can be used to transmit pathogens.

Although it might be more challenging than it sounds, it is time to bridge the gap in the disease surveillance system through a “One Health” approach to understand the dynamics of the past, control the pathogens of the present, and avert more dangerous strains to come. Being able to identify the dynamics of encounters between different ecosystems of pathogen-host associations in this microbe-dominated planet would help identify how they drive the disease arena. Surveillance sites through a “One Health” system will enable us to simultaneously monitor all ID pathogens and save much of the destructive potential, which stems from the fact that they often strike unexpectedly, leaving little time for preparation.

## Figures and Tables

**Table 1:** List of data sets used for the study. It indicates the description of data and source.

Ser.	Data	Description	Details	No. of records	Year	Source
1	GIDEON	Global Infectious Disease and Epidemiology Online Network	It provides infectious diseases of countries and updates yearly	246	2000–2020	https://www.gideononline.com/eboo%20ks/country/infectious-diseases-of-Ghana/
2	SpeciesInteraction_EID2	Host-pathogen interaction	This provides the hosts of a pathogen and the pathogens of a host	245	2014	https://doi.org/10.6084/m9.figshare.1381853
3	IHR	International Health Regulations (2005)	It provides progress made in implementing core public health capacities in averting and responding to the international spread of diseases	13	2010–2019	https://apps.who.int/gho/data/node.main.IHR?lang=en
4	GHS-DHIMS2	Ghana Health Service-District Health Information Management System II	Provides case count data (lab-confirmed and presumed) for infectious diseases from 9,373 health facilities across the country that report through DHIMS2	3	2020	http://chimgh.org
5	F&F	The Health Sector of Ghana Facts and Figures	Yearly performance of the health sector. This provides the top twenty causes of outpatient morbidity reported	20	2002–2017	https://www.moh.gov.gh/facts-figures/
6	Annual reports	Annual reports on Gold Coast and Ghana	It provides the performance of every sector within the country	51	1895–1954	https://libsysdigi.library.Illinois.edu/ilharvest/Africana/Books2011-05/5530214/
7	GSS	Ghana Statistical Service	It provides the names of the 260 districts of Ghana	260	2020	https://statsghana.gov.gh/
8	City Population	Ghana Administrative Division	It provides the administrative divisions of the 260 districts of Ghana	260	2020	https://www.citypopulation.de/en/ghana/admin/

**Table 2:** The transmission routes and their constituents are adapted from GIDEON. None indicates that their transmission route is not part of the categories stated, while unknown indicates that the route of transmission is not known.

Transmission route	Constituents
Direct contact	Bite, sexual contact, breastfeeding, trauma, childbirth, animal scratch, inoculation, blood transfusion, endogenous, and surgery
Airborne or droplet-borne	Respiratory or pharyngeal acquisition, inhalation, air, aerosol from animals and other objects
Fomite	Water, dust, infected secretions, diary and other food products, faecal-oral, vectors, urine, saliva, and contaminated products
None	These transmission routes are not part of the above categories
Unknown	Transmission routes not known

**Table 3:** The transition of IDs that caught global attention, their years of existence and burden in the world, and the year they reached Ghana.

Infectious disease of global proportion	Year of existence and burden in the world	Year it reached Ghana	Reference
Influenza pandemic	1889–1893	1891	[38]
Bubonic plague	1894–1912	1908	[39]
Bubonic plague	1913–1931	1924	[39]
Influenza pandemic	1918–1920	1918	[40]
HIV/AIDS	1981–present	1982	[41]
SARS-CoV-2	2019–present	2020	[42]

**Table 4:** The type of pathogens and reservoirs of infectious diseases in Ghana. The columns indicate the number in percentages of pathogens for each reservoir, and the rows indicate the number in percentages of reservoirs for each pathogen. Bacteria pathogens (column 2) hosted by humans (row 2) were the majority (number = 100 with 43.7%). Protozoa and fungi (rows 16 and 17) serving as reservoirs were represented to have the least pathogens (number = 3 with percentages of 66.7 and 33.3) they host, respectively.

Host	Bacteria (%)	Virus (%)	Parasite (%)	Fungus (%)	Protoctista (%)	Unknown (%)
Humans	43.7	25.8	21.3	8.3	0.4	0.4
Domestic animals	45.1	21.3	22.6	10.4	—	0.6
Mammals (excluding humans)	42.6	26.9	19.4	10.2	0.9	—
Arthropods	61.0	15.3	11.9	11.9	—	—
Plants	71.8	5.1	2.6	20.5	—	—
Primates (excluding humans)	31.4	41.4	20.0	7.1	—	—
Aves	52.6	21.0	15.8	10.5	—	—
Fishes	60.7	7.1	14.3	14.3	3.6	—
Rodents	39.3	35.7	10.7	14.3	—	—
Reptiles	66.7	—	6.7	26.7	—	—
Invertebrates	50.0	—	35.7	14.3	—	—
Worms	100.0	—	—	—	—	—
Amphibians	50.0	16.7	33.3	—	—	—
Marine animals (excluding fish)	50.0	—	16.7	33.3	—	—
Protozoa	66.7	—	33.3	—	—	—
Fungi	66.7	—	—	33.3	—	—
Bacteria	50.0	25.0	—	25.0	—	—

**Table 5:** The breakdown of peer-reviewed surveillance research of endemic or potentially endemic infectious diseases in Ghana from 2000 to 2020. The table indicates for each category the type of district (district, municipal, and metropolitan), the number of pathogens studied, the number of areas the studies were carried out, and the number of times the studies were conducted. *P*: pathogens studied; *A*: areas studied; *T*: number of times the studies were carried out.

Group of pathogen	Total number of pathogens	Number of pathogens studied	District			Municipal			Metropolitan		
			*P*	*A*	*T*	*P*	*A*	*T*	*P*	*A*	*T*
Bacteria	106	29	12	54	125	12	32	64	21	4	53
Virus	63	36	24	41	158	26	32	133	32	5	130
Parasite	56	34	26	107	326	27	74	264	27	6	128
Fungi	24	3	0	0	0	2	3	3	3	3	6
Protoctista	1	0	0	0	0	0	0	0	0	0	0
Unknown	7	0	0	0	0	0	0	0	0	0	0

## Data Availability

The data used for the research findings are available from the corresponding author upon request.

## Abbreviations

AIDS: Acquired immunodeficiency syndrome
COVID-19: Coronavirus 2019
DHIMS2: District Health Information Management System II
EID: Emerging infectious diseases
ERID: Emerging and re-emerging infectious disease
GHS: Ghana Health Service
GHSI: Global Health Security Index
GIDEON: Global Infectious Diseases and Epidemiology Network
GSS: Ghana Statistical Service
HIV: Human immunodeficiency virus
ID: Infectious disease
IHR: International Health Regulations
MERS: Middle East respiratory syndrome
PPMED: Policy Planning, Monitoring, and Evaluation Division
RID: Re-emerging infectious disease
SARS-CoV-2: Severe acute respiratory syndrome coronavirus 2
SSA: Sub-Saharan Africa.

## Supplementary Materials

Supplementary Materials The supplementary materials include the administrative divides and the agroecological zones of Ghana as shown in Figure S1 and S2, respectively. Ghana is divided administratively into 16 regions with 6 metropolitan districts, 107 municipal districts, and 147 districts. The agroecological zones as adapted from [26] shows the dry northern savannah, the humid middle forest rainfall zone, and the southern savannah and mangroves. Figure S1: The demographic and administrative divisions of Ghana. This map shows the 6 metropolitan districts, 107 municipal districts, and 147 districts. Figure S2: The agroecological zones of Ghana. These agroecological zones were adopted from [26].
