# Supplementary material for: Reviewing the Past, Present, and Future Risks of Pathogens in Ghana and What This Means for Rethinking Infectious Disease Surveillance for Sub-Saharan Africa
Source: J Trop Med. 2022 Jul 14;2022:4589007. doi: 10.1155/2022/4589007 (PMC9284326; doi:10.1155/2022/4589007)
Supplement: Supplementary Materials — The supplementary materials include the administrative divides and the agroecological zones of Ghana as shown in Figure S1 and S2, respectively. Ghana is divided administratively into 16 regions with 6 metropolitan districts, 107 municipal districts, and 147 districts. The agroecological zones as adapted from [26] shows the dry northern savannah, the humid middle forest rainfall zone, and the southern savannah and mangroves. Figure S1: The demographic and administrative divisions of Ghana. This map shows the 6 metropolitan districts, 107 municipal districts, and 147 districts. Figure S2: The agroecological zones of Ghana. These agroecological zones were adopted from [26]. [file 4589007.f1.pdf]

Supplementary Information

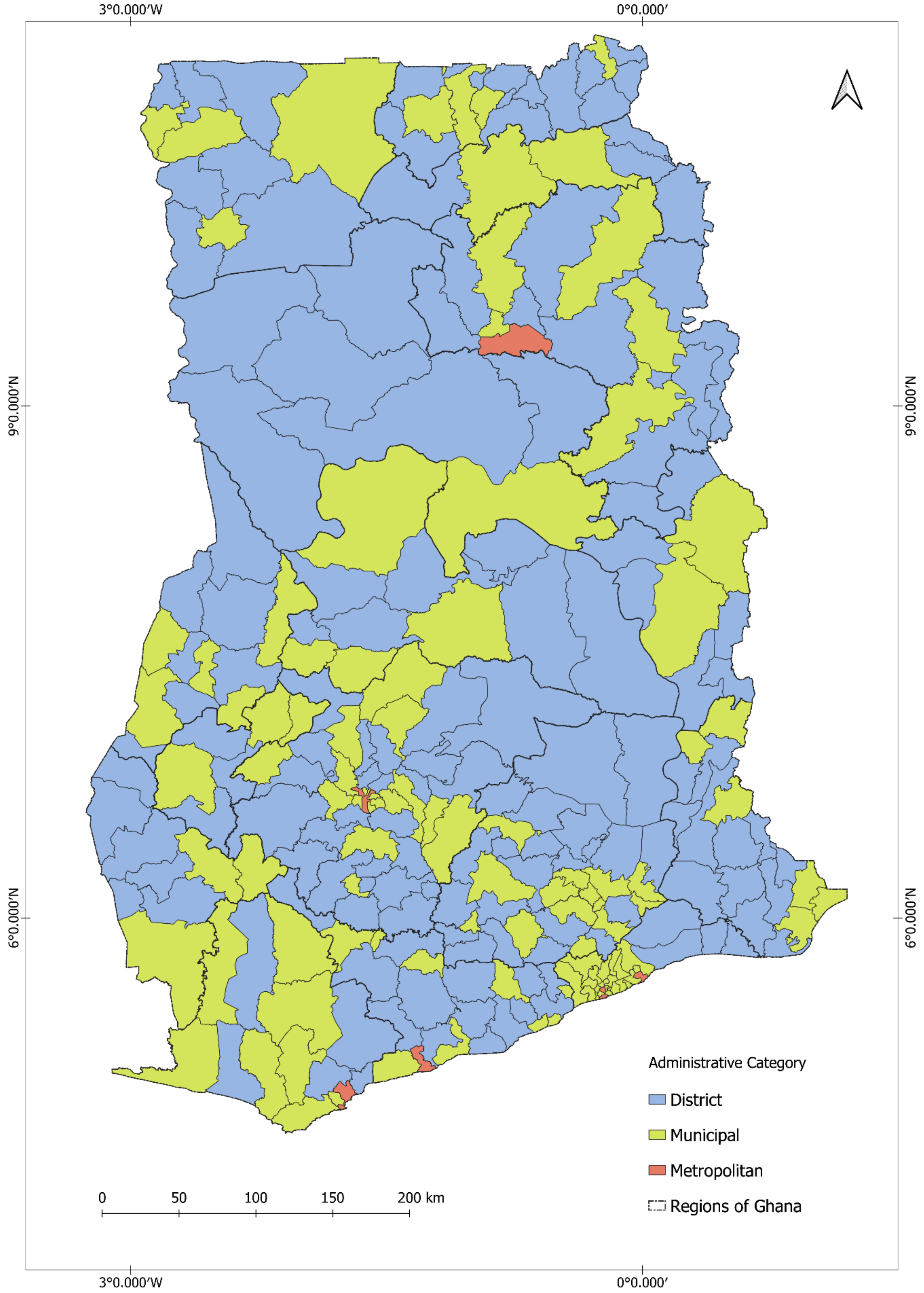

Figure S1. The demographic and administrative divisions of Ghana. This map shows the 6 Metropolitan Districts, 107 Municipal Districts and 147 Districts.

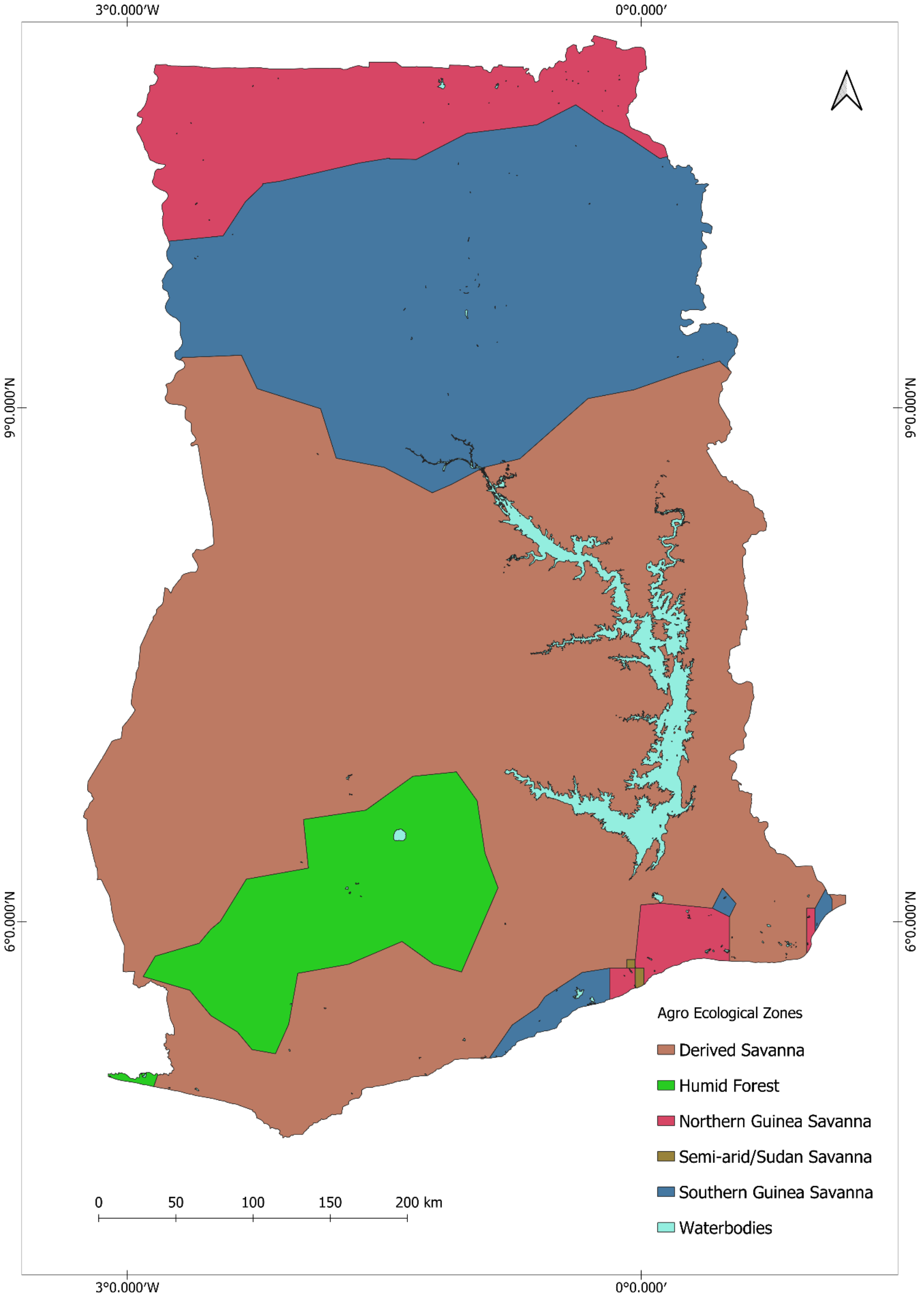

Figure S2. The agroecological zones of Ghana. These agroecological zones were adopted from (World Agroforestry Centre, 2015).
